# Environmental impact of bioplastic use: A review

**DOI:** 10.1016/j.heliyon.2021.e07918

**Published:** 2021-09-03

**Authors:** Ghada Atiwesh, Abanoub Mikhael, Christopher C. Parrish, Joseph Banoub, Tuyet-Anh T. Le

**Affiliations:** a Environmental Science Program, Memorial University of Newfoundland, St. John's, NL A1B 3X7 Canada; b Chemistry Department, Memorial University of Newfoundland, St. John's, Newfoundland A1C 5S7, Canada; c Department of Ocean Sciences, Memorial University of Newfoundland, St. John's, Newfoundland A1C 5S7, Canada; d Fisheries and Oceans Canada, Science Branch, Special Projects, St John's, NL, A1C 5X, Canada; e School of Science and the Environment, Memorial University of Newfoundland, Grenfell Campus, Corner Brook, NL A2H 5G4, Canada; f Environmental Policy Institute, Memorial University of Newfoundland, Grenfell Campus, Corner Brook, NL A2H 5G4, Canada; g Forestry Economics Research Centre, Vietnamese Academy of Forest Sciences, 46 Duc Thang ward, Northern Tu Liem District, Hanoi 11910, Viet Nam

**Keywords:** Bioplastics, Environment, Petroleum-based plastics, Life cycle assessment

## Abstract

Throughout their lifecycle, petroleum-based plastics are associated with many environmental problems, including greenhouse gas emissions, persistence in marine and terrestrial environments, pollution, etc. On the other hand, bioplastics form a rapidly growing class of polymeric materials that are commonly presented as alternatives to conventional petroleum-based plastics. However, bioplastics also have been linked to important environmental issues such as greenhouse gas emissions and unfavorable land use change, making it necessary to evaluate the true impact of bioplastic use on the environment. Still, while many reviews discuss bioplastics, few comprehensively and simultaneously address the positives and negatives of bioplastic use for the environment. The primary focus of the present review article is to address this gap in present research. To this end, this review addresses the following questions: (1) what are the different types of bioplastics that are currently in commercial use or under development in the industry; (2) are bioplastics truly good for the environment; and (3) how can we better resolve the controversial impact of bioplastics on the environment? Overall, studies discussed in this review article show that the harms associated with bioplastics are less severe as compared to conventional plastics. Moreover, as new types of bioplastics are developed, it becomes important that future studies conduct thorough life cycle and land use change analyses to confirm the eco-friendliness of these new materials. Such studies will help policymakers to determine whether the use of new-generation bioplastics is indeed beneficial to the environment.

## 1 Introduction

Plastics have become commonplace manufacturing materials that find applications in a variety of industries, from packaging to the production of toys, from grocery bags to plastic cutlery, from straws to 3D printed rocket nozzles [1, 2, 3, 4, 5]. Chemically, plastics are high molecular weight polymers typically comprising between 1000 to 10000 monomeric repeating units [1, 6, 7]. Conventional petroleum-based synthetic plastics are produced in a series of steps, the first of which is the distillation of crude oil in an oil refinery. This process separates and fractionates the heavy crude oil into groups of lighter components, called segments. Each segment is a mixture of polymeric hydrocarbon chains, which differ in terms of size and structure. One of these fractions, naphtha, is the crucial component needed to generate monomers such as ethylene, propylene, and styrene to produce plastics. These monomers form plastics through polyaddition and/or polycondensation aided by specific catalysts [8, 9]. However, this conversion produces pollutants and greenhouse gases such as carbon dioxide (CO_2_), thus contributing to environmental pollution and global warming [3]. Moreover, several petroleum-based plastics are nonbiodegradable, which leads to their persistence at the site of disposal and harms the environment [10]. Over two recent decades, several studies have suggested alternatives to the conventional petroleum-based plastics. One such alternative is bioplastics, which are polymeric compounds that are both functionally like synthetic plastics and largely environmentally sustainable (Table 1). However, bioplastics are surrounded by myths, for example, all bioplastics are biodegradable and good for the environment. The truth is that some bioplastics may contribute significantly to global warming, pollution, and drastic land use change. Still, while many reviews discuss bioplastics, few comprehensively and simultaneously address the positive and negative dimensions of bioplastic use for the environment. Similarly, some reviews have separately focused on a comparative analysis of bioplastics and conventional fossil fuel-based plastics, specific bioplastics such as polyhydroxybutyrate (PHB), degradation of bioplastics, bioplastic waste management and recycling, and so on, without discussing these concepts in conjunction. Reviewing these concepts, therefore, in relation to one another is important to achieve a comprehensive understanding of the state of the art in the field of bioplastics. Furthermore, recently developed bioplastics, such as chitin-based and mycelium-based bioplastics, have not been significantly discussed in the literature despite their potential industrial value. The primary key to the present review article contributes to address these study gaps. This review, hence, addresses the following questions:

(1) What are the different types of bioplastics that are currently in commercial use or under development in the industry?
(2) Are these bioplastics truly good for the environment?
(3) How can we better resolve the controversial impact of bioplastics on the environment?

**Table 1:** Important terms and their definitions.

Term	Definition
Bioplastics	Plastics that (1) are biodegradable; or (2) may or may not be degradable but are produced from biological materials or renewable feedstock.
Bio-based plastics	Plastics derived at least partly from renewable sources of carbon such as plant matter. Partially bio-based (or hybrid) plastics contain both renewable and conventional fossil fuel-based carbon.
Bio-compatible	Materials that are not harmful to living organisms.
Biodegradable	Biodegradable materials can be broken down into monomeric or polymeric components, including biomass, water and carbon dioxide or methane, via microorganisms. In an industrial context, biodegradable materials are truly ‘compostable’ and can be almost entirely converted into benign trash within a few months in a composter.
Compostable	Compostable materials can be decomposed through artificially controlled biological processes using standard mixtures of microorganisms in industry.
Digesters/Composters	Controlled environments to enable the biodegradation of waste as per set timelines in industry.
Marine-degradable	Plastics, whether fossil fuel-based or bio-based, that can be degraded into carbon dioxide and water in a marine environment by means of heat, light or microorganisms.
Non-toxic/Toxic	Materials with residual constituents, leached components, or degradation products that are harmful to living organisms.
Plastics	Polymeric materials primarily of synthetic or semi-synthetic origin; most commonly derived from fossil fuels.
Renewable source	A resource that can be used and replenished (through natural means) continually, such as biomass.

Before delving into these questions, it is important to understand some common terms (such as ‘bioplastics’, ‘bio-based plastics’, ‘biodegradable plastics’, etc.) that will be used in this article. The need for defining these terms clearly arises from the confusion that has generally existed in bioplastics literature over what they mean. Table 1 summarizes the definitions of such terms in the context of the present review.

## 2 Methodology

**Figure 1:**
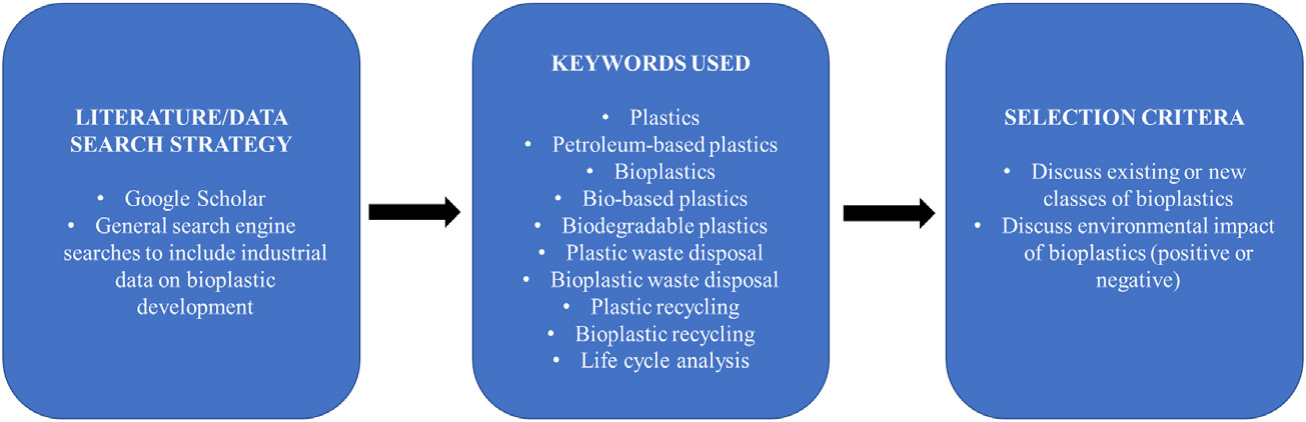
Review methodology.

This review collates and summarises primary data produced and presented by other academic and industrial scholars through their research on bioplastics and their impact on the environment. The following search terms were used in Google Scholar to identify relevant studies to discuss in this study: plastics, petroleum-based plastics, bioplastics, bio-based plastics, biodegradable plastics, plastic waste disposal, bioplastic waste disposal, plastic recycling, bioplastic recycling, life cycle analysis (Figure 1). Industrial research data, such as primary data available on company websites, was not excluded from this review as such data provide information about the competitive, cutting-edge research and development in the field of bioplastic development. To specifically meet the objectives of the present review, only those studies that discussed existing or new classes of bioplastics, and/or their impact on the environment (positive or negative) were included.

The results of this literature review are presented in four sections. The first of these sections, titled ‘Plastics and the environment’, discusses conventional plastics, their degradability, and their impact on the environment. The second section introduces bioplastics such as a way to replace conventional plastics and discusses some of the most important as well as recently developed bioplastics currently in commercial use or industrial testing. The third section elucidates the debate about whether bioplastics or not are good for the environment, presenting both the positive and negative effects of these materials on the environment. The last of the four sections introduces life cycle assessment considered like a means to address the debate around the eco-friendliness of bioplastics, referencing some preliminary analyses published by other researchers.

## 3 Plastics and the environment

The global consumption of plastics has increased over the years, particularly because they are lightweight, resilient, relatively low-priced, and long-lasting. The plastic industry generates approximately 300 million tons of plastics annually, which are used once and discarded after use [11]. Discarded plastic waste, owing to the durability and low degradability of these polymers, may take hundreds to thousands of years to decompose [11]. Moreover, of the total produced quantity of plastics, only 7% is recycled, while about 8% is incinerated and the residual landfilled [12]. The National Academy of Sciences in 1975 assessed that 14 billion pounds of garbage was dumped every year, either buried underground or buried in the oceans. Consequently, oceans and landmass are infested with plastics. In fact, more than 10 million tons of plastic waste is dumped in the oceans alone, so that the majority of anthropogenic debris littering the oceans is composed of human-made plastics. Reports suggest that plastics can now be used as a geological stratigraphic indicator of the Anthropocene era [13, 14, 15, 16]. This anthropogenic debris threatens ocean safety, integrity, and sustainability [17]. Overall, plastic waste contributes to a pressing environmental problem is as yet unsolved.

### 3.1 Why plastics are nondegradable

The production of synthetic plastics, particularly nondegradable ones, is an environmental burden. This is because ‘nondegradable’ plastics take decades or centuries to break down [18]. Nonbiodegradability of certain plastics suggests that their chemical structure cannot be adequately modified by naturally occurring microorganisms, water, carbon dioxide or methane to degrade them [10, 19]. Meanwhile, ‘biodegradable’ plastics are truly compostable materials that can almost entirely be converted into benign trash after a matter of months in a composter [18].

Studies on biological decomposition of plastics by various microorganisms under different environmental conditions have revealed that these decomposition conditions are governed by the physical and chemical characteristics of the type of plastic discarded, such as mobility, crystal structure, molecular weight, functional groups etc. [20]. High molecular weight, high degree of crystallinity, high hydrophobicity as a result of linearity of the polymeric carbon chain backbone, and general insolubility in water are some of the factors that typically reduce the degradability of plastics [20, 21, 22]. Indeed, these are the properties that make the petroleum-based plastics polyethylene and polypropylene nonbiodegradable [10, 22].

Notably, not all petroleum-based plastics are nonbiodegradable. For example, polycaprolactone (PCL) and poly(butylene succinate) (PBS) are both petroleum-based plastics which can undergo microbial degradation [10]. However, the biodegradability of these polymers is affected by their physicochemical properties such as degree of crosslinking, degree of crystallinity, molecular weight and the species of microorganisms used [23]. Indeed, studies have revealed that crosslinked polymers have the lowest rate of degradation, followed by crystalline and then amorphous polymers [23].

### 3.2 How to eliminate plastics

There are many alternatives currently available for reusing and recycling existing plastics, and a significant amount of ongoing research seeks to completely replace plastics with more sustainable alternatives in the future. At the same time, a large amount of plastic waste is already present in the environment and needs to be disposed. Moreover, recycling of plastics has not been effectively adopted. Also, plastics can only be recycled a limited number of times before they become contaminated to the point that they can no longer be used [17].

The challenge of plastic disposal can be addressed in various ways. One way is to convert the plastic discards into energy by incineration [24]. However, this will give rise to large amounts of carbon dioxide and contribute to global warming. A more sustainable means of disposing old plastics is to develop the capability to recycle old plastic materials into new ones. An example is the production of recycled oxy-degradable plastics (synthetic wood) from high-molecular polyethylene to replace wood for discarded garden furniture [25]. Other alternative approaches to plastic recycling include mechanical and chemical recycling. Mechanical recycling permits plastic discards to be used as raw material for other new types of plastic products [26]. When mechanical recycling is not possible, chemical recycling technologies can be used to convert plastic waste into different products through chemical breakdown processes [26]. Chemical recycling of plastic waste involves depolymerization to the constituent monomers achieved through hydrolysis, alcoholysis, glycolysis, ammonolysis, pyrolysis, hydrogenation, and gasification [26]. However, whether recycled plastics are better for the environment can only be determined after knowing if the production of new plastic materials will allow overall reductions in energy expenditure, water use and greenhouse gas emissions [27, 28].

Lastly, another method of eliminating plastic waste is to use it to generate gaseous matter with high hydrogen content or synthesis gas [7]. This is a promising alternative to waste treatment because not only is waste eliminated, but it is also used as fuel.

## 4 Bioplastics

The environmental problems caused by discarded synthetic plastics have paved the way for the search for substitutes. Bioplastics, which are both functionally similar to synthetic plastics and environmentally sustainable, are touted as promising new materials to address these problems. Bioplastics is a term used to refer to plastics that (1) are biodegradable, such as PCL or PBS; or (2) may or may not be degradable but are produced from biological materials or renewable feedstock, such as starch, cellulose, vegetable oils, and vegetable fats [10, 19]. Like any other polymeric material, the degradability of bioplastics is also a factor of their composition, degree of crystallinity and environmental factors, leading to degradation times ranging from several days to several years. For these reasons, the development of biodegradable bioplastics has gained attention in recent years [24, 26, 28, 29].

Based on degradation mechanisms, there are two main categories of biodegradable bioplastics, namely oxo-biodegradable and hydro-biodegradable [30]. Oxo-biodegradable plastics are made of petroleum-based polymers mixed with a pro-degradant additive that catalyzes the plastic's degradation process [31]. The additive is a metal salt (manganese or iron salts), which enhances the abiotic degradation process of the oxo-biodegradable plastic in the presence of oxygen [32, 33]. Presently, oxo-biodegradable plastics are mainly produced from naphtha, a by-product of oil or natural gas [34]. Interestingly, the time taken by biodegradable oxo products to degrade can be ‘programmed’ at manufacture, like the methane or nitrous oxide industrial processes [31]. The degradation of oxo-biodegradable plastics usually takes months to years [32]. On the other hand, hydro-biodegradable plastics decompose hydrolytically at a rate faster than oxo-degradable plastics. These plastics can be converted to synthetic fertilizers. Examples include bioplastics produced from plant sources (such as starch), and polylactic acid (PLA). Forthcoming paragraphs summarize the most recent literature on different types of bioplastics that have been or are currently being developed.

### 4.1 Thermoplastic starch

Starch is a biodegradable, cheap, renewable, easily modifiable biopolymer acquired from renewable plant resources [34, 35]. It consists of two main constituent polymers, amylose, and amylopectin. Amylose is a linear polysaccharide composed of α-D-glucose monomers linked by α-1,4-glycosidic linkages, whereas amylopectin has the same composition but is highly branched through another type of linkage, the α-1,6-glycosidic linkage [36]. It should be noted that starch chains bind together via strong hydrogen bonding, which results in a rigid structure composed of highly ordered crystalline regions [36, 37, 38, 39].

Starch can be formulated into suitable thermoplastic material that can be readily processed into useable forms [39, 40]. Starch's thermal processing involves a change in its microstructure, phase transitions and rheology. Furthermore, starch can be chemically modified and blended with other biopolymers to reduce its brittleness. Starch-based bioplastics are used for packaging materials and for producing food utensils such as cups, bowls, bottles, cutlery, egg cartons, and straws.

### 4.2 Polyhydroxyalkanoates

**Figure 2:**
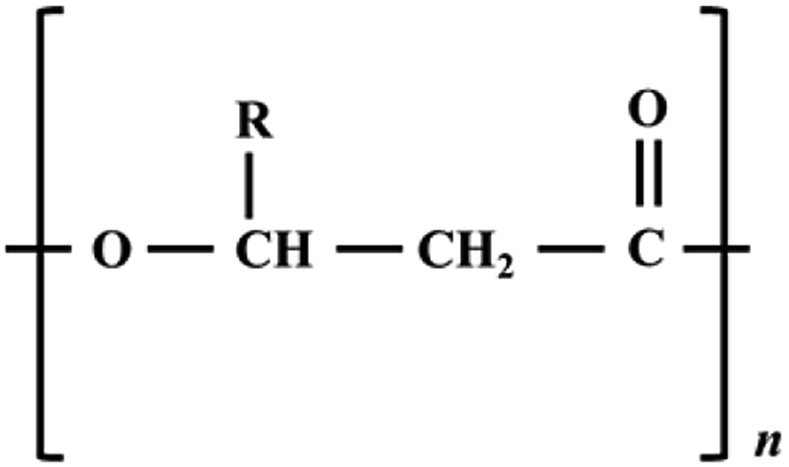
Chemical structure of PHA.

Polyhydroxyalkanoates (PHAs) are a class of bio-based plastics belonging to the polyhydroxyester family of 3-, 4-, 5- and 6-hydroxy alkanoic acids [41]. The general chemical structure of PHA is shown in Figure 2. PHAs are biocompatible, biodegradable, and non-toxic polyesters synthesized by certain bacteria and plants from renewable sources [41]. In particular, PHA can be produced from methane released from feedstock in wastewater treatment facilities, landfills, compost facilities, farms and food processors, waste haulers, bio-refinery operators, and plastic compounders can be used as feedstock for successful, low-cost commercial production of PHA [42, 43]. PHA can also be produced from wood biomass, grass, energy, and crop residues instead of more expensive biomass obtained from edible crops (Renmatix, Pennsylvania, USA) [44]. Renmatix's technology separates biomass from water and uses heat instead of acids, solvents, or enzymes to produce PHA bioplastics in a clean, fast and relatively inexpensive process [42]. The PHA thus produced can be used for commercial purposes, such as bioplastic wraps, shampoo bottles, or polyester fibers that can be combined with natural materials for clothing. PHA bioplastics can be digested naturally by marine microorganisms when they are decomposed into methane and reach the ocean [42]. At the end of its life cycle, the developed bioplastic can be broken down into virgin plastic since it is compostable and marine-degradable [42, 45].

**Figure 3:**
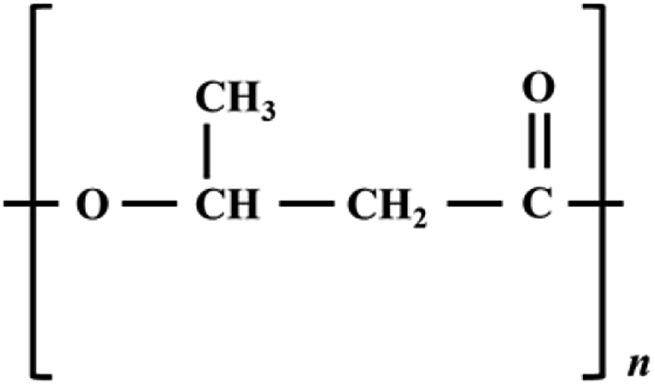
The structure of PHB plastic.

PHB is a widely-used PHA (Figure 3) produced by a variety of microorganisms (such as *Cupriavidus nectar*, *Methylobacterium rhodesianum* or *Bacillus megaterium*) from methane [46, 47, 48]. Methane is first oxidized to methanol via the methane monooxygenase enzyme catalytic pathway [49]. This is followed by methanol dehydrogenase-dependent conversion of methanol to formaldehyde [49]. Methanotrophic bacteria, such as γ-proteobacteria and α-proteobacteria, can further convert formaldehyde to acetyl coenzyme A (Acetyl-CoA) [49, 50]. Acetyl CoA is condensed into the dimer acetoacetyl-CoA, which is then reduced by acetoacetyl-CoA reductase enzyme to form PHB monomer β-hydroxybutyrl-CoA [49]. Finally, β-hydroxybutyrl-CoA is polymerized to PHB via the PHB synthase enzyme [49].

PHB bioplastics are biodegradable, making them an attractive environment-friendly alternative to fossil-based thermoplastics [51, 52]. Melt-processable PHB can be formed by using semi-crystalline thermoplastics produced from the fermentation of renewable carbohydrate feedstock [53]. Moreover, commercial grades of PHB possess properties very similar to fossil fuel produced polypropylene (PP) [54, 55].

Common applications of PHB include disposable tableware articles, soil retention sheathing, waste wrapping, and packaging material. PHB also finds applications in the field of biomedical engineering where it can be spun into surgical sutures and used as drug delivery systems [55].

### 4.3 Polylactic acid

**Figure 4:**
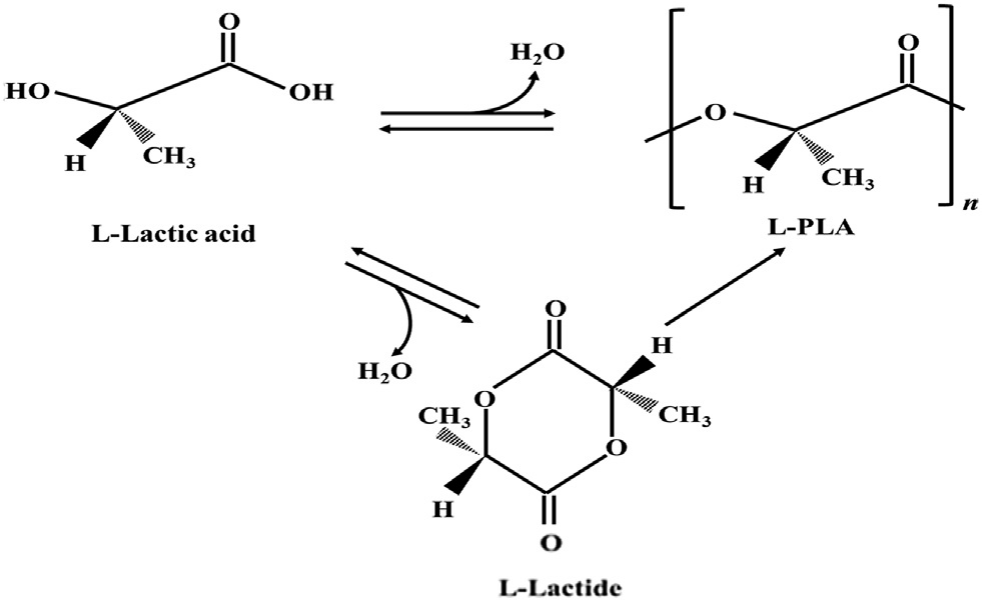
Polylactic acid (PLA) hydrolysis.

Polylactic acid (PLA) is a thermoplastic aliphatic polyester obtained by polymerizing lactic acid from renewable resources, such as corn starch, tapioca roots, chips or starch, and sugarcane [56]. PLA is used mainly in the food industry to prepare disposable tableware articles like drinking cups, cutlery, trays, food plates, food containers and packaging for sensitive food products. However, PLA bioplastics are too fragile and cannot be used for other packaging manufacturing processes. For this reason, PLA needs additives to make it more durable [57]. Notably, PLA is the most biodegradable thermoplastic, typically degrading via hydrolysis (Figure 4) [58].

Several commercial grades of PLA are specifically designed for processes such as thermoforming and extrusion/injection moulding [59]. It can also be used for soil retention sheathings, agriculture films, waste shopping bags, and the use of packaging material [58]. Furthermore, PLA can be converted into fibers by spinning and used to manufacture woven, disposable and biodegradable fabric articles such as disposable garments, feminine hygiene products, and diapers [43, 58].

### 4.4 Bioplastics produced by cyanobacteria through photosynthesis

Recent studies have described the production of bioplastics by using cyanobacteria blooms that use sunlight to produce chemicals through photosynthesis [60]. Instead of feeding sugar from corn or sugarcane to plastic-producing bacteria, advances have been made to improve the cyanobacteria to produce plastics naturally by using their self-synthesized glucose. Cyanobacteria can convert glucose to acetyl-CoA, which, as explained earlier, is then converted to acetoacetyl-CoA, followed by β-hydroxybutyryl-CoA and finally, PHB [60]. Moreover, it has been shown that it is also possible to produce polymers from genetically engineered cyanobacteria that feed on sugars, a method that could replace fossil-fuel-based processes [61, 62, 63]. Overall, cyanobacterial species such as *Scytonema geitleri Bharadwaja*, when stressed, store the intracellular poly-β-hydroxybuyrate granules for energy and carbon reserves inside their cells [64]. The biodegradable and eco-friendly PHB can then be gathered and used to form biocompatible thermoplastics [63].

However, researchers have pointed out a possible issue with bioplastic production that relies on feeding plastic-producing bacteria with large quantities of sugars obtained from natural crops. Since the natural crops are used as food to sustain people and animals, we risk compromising the competing balance for the limited agricultural resources [65]. As a potential solution for this issue, a recent study has demonstrated the development of finely tuned cyanobacteria of the *Spirulina* strain, which can constantly produce sugar and leak it into the surrounding saltwater, which contains natural bacteria [66]. These bacteria usually feed off the leaked sugar and convert it to produce bioplastic. This means that the cyanobacteria create sugar during photosynthesis, which is food for the natural bacteria that converted it into bioplastics [66].

**Figure 5:**
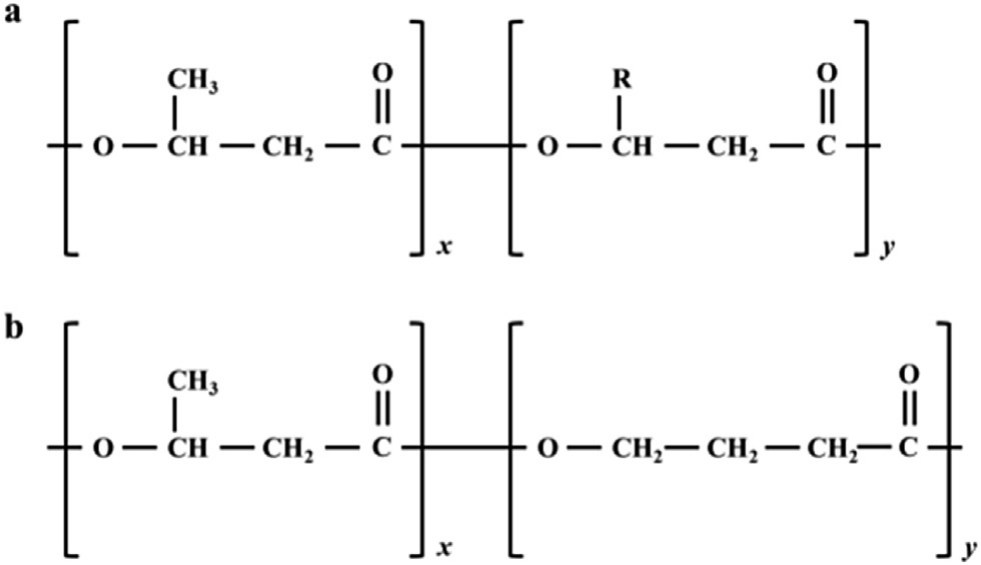
(a) Poly-hydroxybutyrate copolymers. (b) Poly (3-hydroxybutyrate-co-4hydroxybutyrate) (PHB4B).

Promising new strategies involving genetic engineering of cyanobacteria have also been reported to produce small substrate chains like poly (3-hydroxybutyrate-co-3-hydroxyvalerate) PHBV and poly (3-hydroxybutyrate-co-4-hydroxybutyrate) PHB4B, and PHBHx copolymers containing 3-hydroxyl hexanoate units [60]. This involves the use of a mixture of substrates, such as glucose and valerate, to cause the formation of random copolymers [60]. Hence, when these substrates are alternately bonded during copolymerization, it is possible to obtain PHA block copolymers synthesized by bacteria [67]. The chemical structures of these copolymers are shown in Figure 5.

### 4.5 1,2-, 1,4- and 2,3-butanediol bioplastics

**Figure 6:**
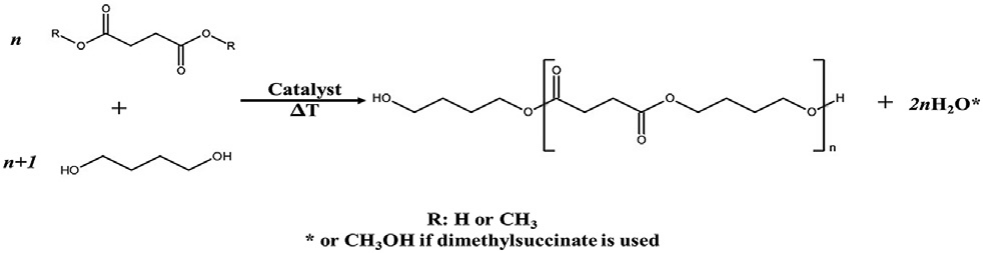
Poly (1,4-butylene succinate) (PBS).

Butanediol (BDO) is an industrial chemical used as a solvent and building block in bioplastics, elastic fibers, and polyurethanes [68]. BDO contains terminal, primary hydroxyl groups which allow it to be used as a cross-linking agent for the synthesis of thermoplastic urethanes, polyester plasticizers, paints and coatings, copolyester hot melt and solvent-borne adhesives [69]. In polyurethane applications, 1,4-BDO is primarily used as a component of polyesters or as a chain extender. Bioplastics formed from BDO are completely biodegradable. An example is poly (1,4-butylene succinate) (PBS). PBS, which typically exists behaves as a semi-crystalline thermoplastic, is chemically synthesized from succinic acid and 1,4-BDO (Figure 6).

The mechanical properties of PBS are comparable to that of widely used high-density polyethylene and isotactic polypropylene [70, 71, 72]. Moreover, it is relatively more cost-effective compared to other biopolymers such as PLA, PBAT, and PHB [70, 71, 72]. As such, it is used for a variety of applications such as disposable food packaging, mulch film, plant pots, hygiene products, fishing nets, and fishing lines [70, 71, 72]. It can also be utilized as a ‘matrix polymer’ or in combination with other biopolymers such as PLA [70, 71, 72].

The key monomer for PBS, namely, 1,4-BDO, is currently produced through feedstocks derived from oil and natural gas [73]. Furthermore, it is also possible to synthesize 1,4-BDO via direct biocatalytic routes from renewable carbohydrate feedstocks (glucose and sucrose) [73]. It has also been found that an engineered *Escherichia coli* host enhances the anaerobic operation of the oxidative tricarboxylic acid cycle, thereby generating reducing power to drive the BDO pathway [74]. *E. coli* produce BDO from glucose, xylose, sucrose, and biomass-derived mixed sugar streams. The creation of such engineered bacteria has allowed for a systems-based metabolic engineering approach to strain design and development that can enable new bioprocesses for commodity chemicals that are not naturally produced by living cells.

In addition to 1,4-BDO, it has been established that 2,3-butanediol (2,3-BDO) is an excellent bio-based chemical possessing important industrial applications. 2,3-BDO has been used extensively for synthetic rubber precursor, food additives, and cosmetics. As in the case of 1,4-BDO, *E. coli* has been metabolically engineered to promote the production of 2,3-BDO by expressing the *Bacillus subtilis alsS*, *alsD*, and *ydjL* genes encoding α-acetolactate synthase, α-acetolactate decarboxylase, and acetoin reductase/2,3-butanediol dehydrogenase, respectively, along with *Deinococcus radiodurans dr1558* gene encoding a response regulator [75, 76]. In another study, USA-based Genomatica, Inc. developed a commercial, bio-based processes to manipulate *E. coli* to produce bio-butanediol (Bio-BDO) directly [77]. This bio-butanediol (Bio-BDO) chemical can be used to create a wide range of products: from spandex to car bumpers, in a more energy-efficient way and without oil or natural gas [77].

### 4.6 Seaweed polysaccharide bioplastics

Seaweeds are excellent candidates for the production of bioplastics [78]. Seaweeds possess the ability to grow in a wide range of environments, which simplifies their cultivation in the natural environment [79]. Using seaweeds for bioplastics production can minimize the impact on the food chain [78, 80]. Furthermore, seaweed-based bioplastics are chemical-independent [78, 80].

The most commonly used seaweed types in industry contain polysaccharides such as agar, alginate, carrageenan, galactans, and starch [78]. These polysaccharides consist of mannuronic and guluronic acid residues [43, 81]. The seaweed polysaccharide backbones are frequently functionalized with various substituent sulphate and methoxyl groups, which impart negative charge to them [82]. This allows them to interact to variable extent with cations, resulting in the formation of gels [82]. These gels have properties that cover a wide range of industrial applications required by all thermo-mechanical bioplastics [82].

Seaweed polysaccharides are extracted from dried and ground seaweeds by following a hot extraction method [78]. This is followed by a two-step purification process, the first of which involves the removal of dense cellulosic contaminants by centrifugation and subsequent filtration, and the second one involves the concentration of the purified mixture by allowing the water to evaporate [78]. From the enriched mixture, potassium chloride can be added to cause gelation of seaweed polysaccharides [78]. Alternatively, isopropyl alcohol can be used to cause precipitation of the polysaccharides [78]. The concentrated mass of polysaccharides can be frozen and freeze-dried to be used in the manufacturing of bioplastics [78]. An example is the production of thermoplastic starch from seaweed starch, as discussed previously in Section 3.1.

Seaweed polysaccharides can be useful in various food industry applications such as texture modification, colloidal stabilization, fat reduction and shelf-life extension [82]. It is also possible to produce biodegradable water bottles made from seaweed [76, 78]. Other applications include lenses, coatings for telephones and DVDs and packaging materials [83].

### 4.7 Fungal mycelium-based bioplastics

Evocative, a New York-based company, has used mycelium – vegetative fungal extensions that give rise to mushrooms – to make plastic-like materials for biodegradable packaging and tiling [84, 85]. Mycelium is composed of polysaccharides, chitin, proteins and lipids, which together result in adequate mechanical properties for this biomaterial to be used in a range of industrial applications [86]. The mushroom-producing mycelium provides for a fibrous biomaterial that can be combined with agricultural by-products (such as the peel of the seeds and the corn stalk) to make composite materials for industrial use [84, 85, 86, 87]. This new material is being used by IKEA company which, to fulfill its commitment to sustainable innovation, has decided to use mushroom-based packing that eliminates the need for other wasteful materials [88].

### 4.8 Bioplastics from crab shells and tree discards

Jie Wu (2014) created a novel bioplastic derived from crab shells and tree fibers that can be used as an alternative for the flexible plastic packaging used to keep food fresh [89]. Multiple layers of chitin from crab shells and cellulose from trees were sprayed to form a flexible film similar to plastic packaging film. This new bioplastic was compared to polyethylene terephthalate (PET), the most common petroleum-based plastic used as transparent packaging. The study revealed that this new packaging could be more effective and safer to contain liquids and foods [90, 91]. In comparison to fossil fuel-based PET plastics, the novel bioplastic material showed a 73% reduction in oxygen permeability, thereby enabling food to stay fresh for longer [92].

## 5 Are bioplastics good or bad for the environment?

Bioplastics are emerging to be highly controversial when it comes to determining their impact on the environment. While bioplastics are often hailed as excellent alternatives to conventional plastics, they are also associated with shortcomings [93]. Let us consider the case of biodegradable bioplastics. Biodegradable bioplastics can decompose into natural materials through microbial mechanisms and blend harmlessly into the soil [94, 95]. This decomposition process is aided by water and/or oxygen. For example, when a cornstarch-derived bioplastic is composted, the cornstarch molecules slowly absorb water and swell up when buried. This causes the starch bioplastic to break apart into small fragments that can then be easily digested by bacteria [94, 96, 97, 98, 99]. However, some low-degrading or nondegradable bioplastics only break-down at high temperatures or when treated in municipal composters or digesters [100, 101, 102]. Moreover, some biodegradable plastics can only degrade in specific active landfill sites under certain definite and tried conditions [103]. Decomposition during composting produces methane gas, a greenhouse gas many times more potent than carbon dioxide [104, 105]. This greenhouse gas contributes to the problem of global warming [106].

Furthermore, producing bioplastics from plants such as corn and maize requires repurposing of land for producing plastic instead of fulfilling food requirements [107]. A recent statistical study revealed that almost a quarter of the agricultural land producing grains is used to produce biofuels and bioplastics. As more agricultural land gets used to produce biofuels and bioplastics, there may be a significant rise in food prices, affecting the economically weaker sections of the society [108].

Moreover, a recent study, which compared seven traditional plastics, four bioplastics, and one made from both fossil fuel and renewable sources, determined that bioplastic production resulted in greater amounts of pollutants, owing to the fertilizers and pesticides employed in cultivating the crops, in addition to the chemical processing needed to turn organic material into the plastic [109]. It was also found that bioplastics contribute more to ozone depletion than traditional fossil fuel-derived plastics [110]. Furthermore, it has been found that bio-based PET, a hybrid bioplastic, is a potential carcinogen and also has pernicious toxic effects on earth ecosystems [111, 112].

At the same time, bioplastics also have eco-friendly characteristics. For example, production of PLA saves two-thirds of the energy needed to make traditional plastics [51]. Moreover, it has been scientifically established that during the biodegradation of PLA bioplastics, there is no net increase in carbon dioxide gas [58]. This was evidenced by the fact that the plants from which they were produced absorbed the same amount of carbon dioxide when they were cultivated as was released during their biodegradation [58, 113]. Notably, PLA emits 70% less greenhouse gases when it degrades in landfills [30]. Other studies have also found that substituting traditional plastic with corn-based PLA bioplastics can reduce greenhouse gas emissions by 25% [110, 112]. Such examples provide assurance that the future production of new bioplastics can be accomplished by using renewable energy while substantially reducing greenhouse gas emissions.

## 6 Life cycle analysis – a way to address the controversy around the eco-friendliness of bioplastics

To comprehensively compare bioplastics with conventional plastics, it is crucial to evaluate bioplastics' environmental impact from the initial production, utilization, and finally to disposal [114, 115]. The most important tool to evaluate the environmental impact of bioplastics and/or conventional plastics is life cycle assessment (LCA) or cradle-to-grave analysis, a process that can help determine the overall impact of a bioplastic on the environment at each stage in its life cycle [115, 116]. This signifies that the whole life of this industrial product is evaluated, starting from the raw material extraction to the various stages of materials processing, manufacture, distribution, and use [116]. An LCA impact study involves the assessment of global warming, human toxicity, abiotic depletion, eutrophication and acidification [117, 118]. In addition, when conducting the LCA, it is essential to consider Land Use Change (LUC)-related emissions and the cost and benefits of bioplastic disposal [119]. LUC is a guide to consider when land is converted to spaces for composting, biofuel feedstock production or other uses [120].

It is essential to understand the LCA of different bioplastic composting, recycling, and disposal scenarios. Indeed, a meticulously performed LCAs can serve as an important reference material for policymakers [121]. For example, numerous protocols have been established to conduct LCA/cradle-to-grave studies on PLA bioplastics currently in the market [122]. These studies involve comparisons of their LCA with that of fossil-fuel plastics such as polyethylene and PET [123]. For instance, a recent study revealed that there was a significant reduction in greenhouse gases when manufactured bottles were made by subsisting 20% of the PET bottles with PLA bottles [124]. This study was carried out by using the Intergovernmental Panel on Climate Change (IPCC) method and a LCA cradle-to-grave study [124, 125, 126]. Another study, using the Global Warming Potential (GWP) guide in which the greenhouse gas emission was measured in kg of CO_2_ equivalents, showed that it was possible to reduce greenhouse gas emissions by substituting petroleum-based plastics with bioplastics [127, 128]. Additional, separate LCAs for other bioplastics can also provide such valuable data.

LCA also provides an important means of identifying the best method of bioplastic waste management and disposal. For example, LCA has revealed that incineration or landfilling of bioplastic products is not a useful alternative [94, 129]. A plausible solution to bioplastic waste management problem was confirmed by adhering to the LUC emissions principle, which established the reliability of bioplastics as an excellent replacement for petroleum-based plastics [130, 131]. Compared to conventional petroleum-derived plastics, the use of PLA and thermoplastic starch significantly reduces carbon dioxide emissions, in the case of the former, by 50–70% [132]. Similarly, bio-urethanes and poly (trimethyleneterephthalate) (PTT) have respectively 36% and 44% lower greenhouse gas emissions than their petroleum-derived counterparts [132]. However, to continue the smart management of bioplastic wastes, it has been proposed that the reduction of greenhouse gas emissions must reach zero LUC emissions [119, 130]. Future studies should focus on conducting individual LCAs for the ever-growing range of bioplastics, many of which have been discussed earlier in this review.

## 7 Conclusion

A variety of bioplastics have been developed to address environmental issues associated with conventional petroleum-derived plastics – from well-known and well-studied biodegradable and/or bio-based plastics like PHB, PCL and PLA to recent additions such as mycelium-based and chitin-based biopolymers. Importantly, however, bioplastics are associated with some shortcomings. It should be understood that similar to petroleum-based plastics, some bio-based plastics cannot be recycled. Consequently, many biodegradable bioplastics end up in landfills, which decompose gradually and produce methane gas. For these reasons, people are starting to believe that bioplastics should be used only when needed, with tailor-made properties. However, it is important that we weigh these environment-related shortcomings of bioplastics against the harms caused by conventional plastics. Studies, including several discussed in the present review article, show that the harms associated with bioplastics are still less severe when compared to conventional plastics. Moreover, as new types of bioplastics such as those discussed in this article keep becoming developed by academic and industry-oriented researchers, it is possible that the drawbacks of currently used bioplastics can be addressed adequately. In order to confirm the eco-friendliness of these new bioplastics, future studies should conduct thorough LCAs and LUC analyses. Such studies will help policymakers to determine whether the use of new-generation bioplastics is indeed beneficial to the environment.

## Declarations

### Author contribution statement

All authors listed have significantly contributed to the development and the writing of this article.

### Funding statement

This research did not receive any specific grant from funding agencies in the public, commercial, or not-for-profit sectors.

### Data availability statement

No data was used for the research described in the article.

### Declaration of interests statement

The authors declare no conflict of interest.

### Additional information

No additional information is available for this paper.
